# Rod and Cone Coupling Modulates Photopic ERG Responses in the Mouse Retina

**DOI:** 10.3389/fncel.2020.566712

**Published:** 2020-09-25

**Authors:** Yichao Li, Ethan D. Cohen, Haohua Qian

**Affiliations:** ^1^Visual Function Core, National Eye Institute (NEI), National Institutes of Health, Bethesda, MD, United States; ^2^Division of Biomedical Physics, Office of Science and Engineering Labs, Center for Devices and Radiological Health, Food and Drug Administration, Silver Spring, MD, United States

**Keywords:** photopic ERG, mouse, gap-junction, rod-cone interaction, photoreceptor, light-adaptation, rd10, retinal degeneration

## Abstract

Light adaptation changes both the sensitivity and maximum amplitude (R_max_) of the mouse photopic electroretinogram (ERG) b-wave. Using the ERG, we examined how modulation of gap junctional coupling between rod and cones alters the light-adapted ERG. To measure changes, a b-wave light adaptation enhancement factor (LAEF), was defined as the ratio of R_max_ after 15 min light adaptation to R_max_ recorded at the onset of an adapting light. For wild-type mice (WT), the LAEF averaged 2.64 ± 0.29, however, it was significantly reduced (1.06 ± 0.04) for connexin 36 knock out (Cx36KO) mice, which lack electrical coupling between photoreceptors. Wild type mice intraocularly injected with meclofenamic acid (MFA), a gap junction blocker, also showed a significantly reduced LAEF. Degeneration of rod photoreceptors significantly alters the effects of light adaptation on the photopic ERG response. Rd10 mice at P21, with large portions of their rod photoreceptors present in the retina, exhibited a similar b-wave enhancement as wildtype controls, with a LAEF of 2.55 ± 0.19. However, by P31 with most of their rod photoreceptors degenerated, rd10 mice had a much reduced b-wave enhancement during light-adaptation (LAEF of 1.54 ± 0.12). Flicker ERG responses showed a higher temporal amplitude in mesopic conditions for WT than those of Cx36KO mice, suggesting rod-cone coupling help high-frequency signals to pass from rods to cone pathways in the retina. In conclusion, our study provides a novel method to noninvasively measure the dynamics and modulation by the light adaptation for rod-cone gap junctional coupling in intact eyes.

## Introduction

The electroretinogram (ERG) is a widely used non-invasive tool to objectively measure the visual function of the eye (Heckenlively and Arden, 2006; Pinto et al., 2007; Weymouth and Vingrys, 2008). Most mammalian retinas contain two types of photoreceptors: rods and cones, which differ in their light sensitivity and response kinetics. In fully dark-adapted animals, dim light stimulation only activates rod photoreceptors which can be used to study rod-mediated retinal pathways, while cone-mediated retinal pathways are commonly studied using an adapting background light to saturate the rod photoreceptors. It is well-known that light adaptation alters cone-mediated photopic ERG responses (Peachey et al., 1992a,b, 1993; Ekesten et al., 1999; Bui and Fortune, 2006). In particular, the maximal amplitude of the photopic b-wave gradually increases during light-adaptation. Such light adaptation-induced growth of photopic ERG responses has been observed in several species, including humans and mice (Burian, 1954; Armington and Biersdorf, 1958; Gouras and MacKay, 1989; Peachey et al., 1993). However, the mechanism of how light adaptation induces changes in the ERG response is still debated (Alexander et al., 2006). Recently, it has been postulated that decoupling of gap junctions between the rod and cone photoreceptors in the retina could contribute to the enhancement of photopic ERG during light adaptation (Heikkinen et al., 2011; Bush et al., 2019).

In this study, we investigated the effect of light adaptation on the mouse photopic ERG and the role of gap junction decoupling. In the mammalian retina, connexin 36 forms gap-junctions between photoreceptors that mediate rod-cone coupling (Bloomfield and Völgyi, 2009; Zhang et al., 2015; Bolte et al., 2016; Jin and Ribelayga, 2016). To test the hypothesis that changes in the ERG amplitude during light adaptation reflect modulation of rod-cone coupling in the retina, we recorded photopic ERG responses in connexin 36 knock out (Cx36KO) mice, and in eyes injected with meclofenamic acid (MFA), a gap junction blocker. Besides, we also followed the photopic ERG response amplitudes with age in rd10 mice during their progressive loss of rod photoreceptors.

Rod-cone coupling serves as the secondary pathway for the rod signal in the mammalian retina (Deans et al., 2002; Völgyi et al., 2004; Fain and Sampath, 2018). This secondary rod pathway activates a subgroup of retinal ganglion cells and mediates vision in mesopic conditions (Völgyi et al., 2004). In this study, we used flicker ERG to investigate the functional benefit of rod-cone coupling in the mammalian retina. We have shown previously that the frequency-response relationship for ERG responses can be determined by the harmonic responses to a single pulse flicker stimulus (Qian and Shah, 2011). Pulse flicker ERG response recorded from wildtype and Cx36KO mice indicated an enhanced signal from photoreceptors to the inner retina through rod-cone coupling under mesopic conditions.

## Materials and Methods

### Experimental Animals

All procedures involving animals were conducted under an approved NIH animal care protocol and following ARVO guidelines on the humane use of animals in ophthalmic and vision research. Connexin36 knock out (Cx36KO) mice were created from the C57BL6 strain by Dr. David Paul (Deans et al., 2001) and maintained by Dr. Jeffrey Diamond’s group at National Institute of Neurological Disorders and Stroke, NIH. C57BL/6J (WT) and rd10 mice were obtained from Jackson Labs (Bar Harbor, ME, USA). All mice were kept in regular animal housing under a 50 lux 14:10 h light/dark cycle.

### Electroretinogram Recording

All procedures were performed under dim red light. Mice were dark-adapted over-night (~20 h) and anesthetized by i.p. injection of ketamine (100 mg/kg)/xylazine (6 mg/kg) mixture. Pupils were dilated with1% tropicamide and 0.5% phenylephrine. Animals were placed on a heating plate to keep body temperature at 37°C. Photopic ERG responses were recorded using an Espion E2 system (Diagnosys) connected with either a regular colordome with four LEDs (blue, 455 nm; green, 516 nm; amber, 595; and red, 636 nm) or an UV colordome with blue LED replaced by an UV LED (367 nm). Responses were recorded using a gold loop wire electrode placed at the center of the cornea, a reference electrode in the mouth, and a ground electrode at the tail. Photopic ERG were captured at 0, 5, 10, and 15 min after the onset of the adaptation light (green, 20 cd/m^2^) to a series of increasing flash (<4 ms) intensities (0.3, 1, 3, 10, 30, and 100 cd.s/m^2^ for green flashes, 338, 1.13 × 10^3^, 3.38 × 10^3^, 1.13 × 10^4^, 3.38 × 10^4^, 1.13 × 10^5^ photons/μm^2^ for UV flashes). Pulse flicker ERGs were recorded with a chain of 6 Hz green flashes delivered at 0.001, 0.002, 0.005, 0.01, 0.02, 0.05, 0.1, 0.2, 0.5, 1, 2, 5, and 10 cd.s/m^2^.

### Intravitreal Injection

Intravitreal injections were performed according to a published protocol (Qian et al., 2008). All procedures were performed under dim-red light. Dark-adapted mice were anesthetized by intraperitoneal injection of ketamine (100 mg/kg) and xylazine (6 mg/kg), and one drop of 0.5% tetracaine topical anesthetic was applied to the cornea. One microliter of the drug (MFA, 200 μM) was injected into the mouse vitreous body. Injections were done through the sclera on the nasal side of the eye approximately 1 mm posterior to the limbus with a 10-μl Nanofil syringe with a removable 35-gauge needle (World Precision Instruments, Inc., Sarasota, FL, USA). Intravitreal concentrations of MFA were estimated to be 10 μM by assuming a complete mixture and the vitreal volume to be ~20 μl for a mouse eye (Wang et al., 2015). Phosphate buffer saline (PBS) was used as an injection control. ERG recordings were performed 2 h after injection with animals kept in dark.

### Data and Statistic Analysis

ERG responses were recorded from both eyes of mice, and averaged values were used for analysis. Intensity-response data were fit with a Naka-Rushton equation:

$$R=R_{max}\frac{I^{n}}{I^{n}+K^{n}}$$

where R_max_ is the maximal response amplitude, *I* is the flash intensity, *n* is the Hill coefficient, and K is the half-saturation constant. Oscillatory potentials (OP) were isolated with a band-pass (40–200 Hz) digital filter (Ramsey et al., 2006). The amplitudes of each harmonic component for pulse flicker ERG responses were derived from a discrete Fourier transform using the Matlab Signal Processing Toolbox (The Mathworks, Boston, MA, USA) from each 10 s recording of the ERG waveform (Shah et al., 2010). Data were expressed as mean ± SEM. Statistics (*t*-test and ANOVA) were performed with Prism (Version 8, GraphPad Software, San Diego, CA, USA).

## Results

### Effects of Light Adaptation on Mouse Photopic ERG

**Figure 1:**
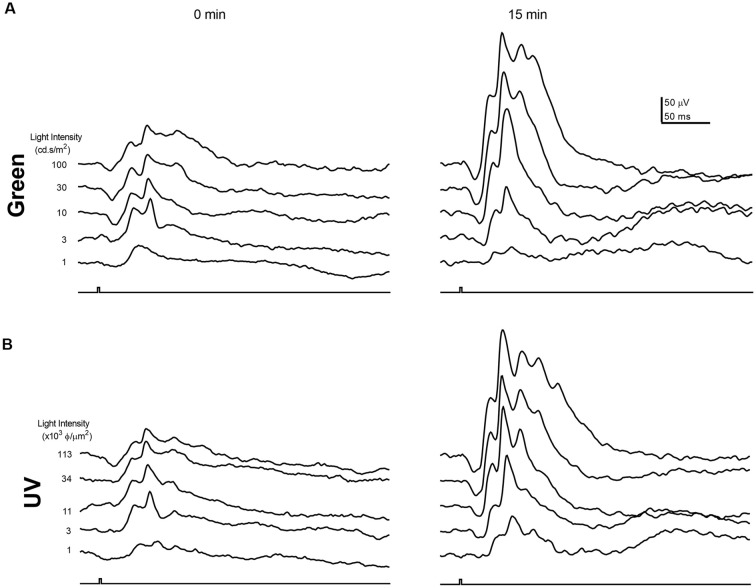
Examples of photopic Green and UV flash electroretinogram (ERG) responses during light adaptation. Example waveforms of the normal mouse photopic ERG elicited by green **(A)** and UV **(B)** flashes immediately (0 min) and 15 min after the onset of the adapting background light. Responses for green and UV flashes were recorded from the same mouse eye.

**Figure 2:**
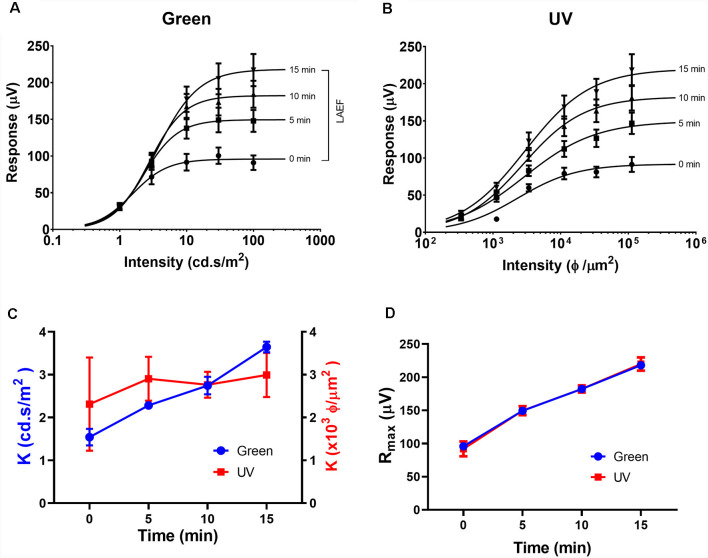
Effects of light adaptation on Green and UV ERG responses. Averaged intensity-response relations for green **(A)** and UV **(B)** responses in normal mice (*n* = 5) elicited at 0, 5, 10, and 15 min after the onset of adapting light. Data were fit with a Naka-Rushton equation. The light adaptation enhancement factor (LAEF) is defined as the ratio of b-wave amplitudes recorded after 15 min light adaptation to those observed at the onset of adapting light (shown as a bracket in **A**). **(C)** Effects of light adaptation on the sensitivity factor (K) for responses elicited by green (left axis) and UV (right axis) flashes. **(D)** Effects of light adaptation on the maximal response amplitude (R_max_) for responses elicited by green and UV flashes.

As the circadian rhythm modulates the photopic ERG and photoreceptor coupling (Cameron and Lucas, 2009; Sengupta et al., 2011; Jackson et al., 2012; Zhang et al., 2015), we performed the ERG recording at the same time of day (i.e., 1 pm) for all the experiments described in this study. Figures 1, 2 illustrate the effects of rod-saturating background light on the mouse photopic ERG responses. As the mouse retina contains both short-wavelength (UV) sensitive and mid-wavelength (green) sensitive cone pigments, we used both UV and green light to probe cone-mediated ERG responses. Figures 1A,B show examples of the ERG waveform elicited by green and UV flashes, respectively, at the onset of adapting background light (green 20 cd/m^2^) at 0 min (left panels) and after 15 min of light adaptation (right panels). The summarized intensity-response relations for b-wave amplitudes elicited by the green and UV flashlights are shown in Figures 2A,B, respectively, at 0, 5, 10, and 15 min after the onset of adapting light. Light adaptation changes both the sensitivity and maximum amplitude of the mouse photopic ERG. The continuous curves were fitted to the Naka-Rushton equation, with the half-saturation values of K and R_max_ plotted against light adaptation time in Figures 2C,D, respectively. For values of K, background adaptation progressively reduced the sensitivity to the green flash (*p* < 0.0001, one-way ANOVA), and 15 min light adaptation reduced sensitivity to the green flash by 2.4 ± 0.5 fold (*n* = 5). On the contrary, the sensitivity to the UV flash is relatively unchanged by the adapting green background (*p* = 0.89, one-way ANOVA). On the other hand, the same light adaptation produced similar enhancements (*p* = 0.92) of the maximum amplitude (R_max_) of the mouse photopic ERG to green flashes (2.3 ± 0.3 fold) as to UV flashes (2.4 ± 0.4 fold, *n* = 5). The a-wave amplitudes of the photopic ERG also increased with light adaptation (green flash 1.79 ± 0.18 fold and UV flash 1.48 ± 0.10 fold). However, the a-wave of the photopic ERG contains multiple components with a large contribution from OFF-bipolar cells in the mammalian retina (Bush and Sieving, 1994; Frishman, 2006). Also, the amplitudes of the photopic a-wave are small which could get contaminated with the noise in the recording. For these reasons, we focused on analyzing the photopic b-wave in this study.

### Effects of Gap-Junction Coupling on Mouse Photopic ERG

**Figure 3:**
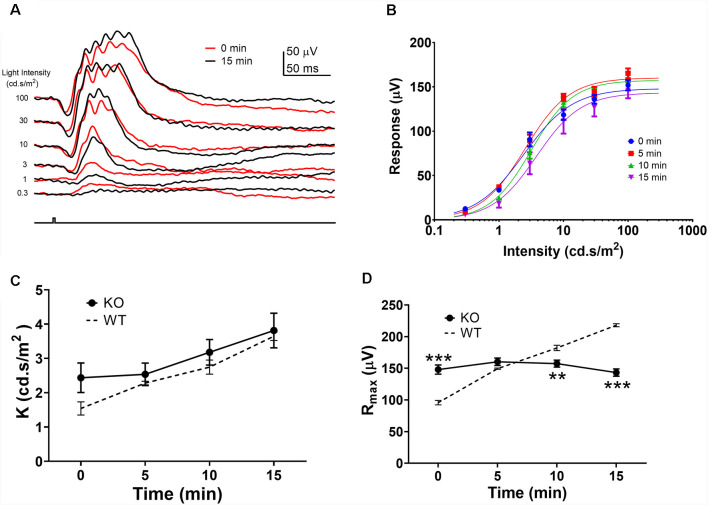
Effects of light adaptation on photopic ERG responses from Cx36ko mice. **(A)** Examples of the photopic ERG waveform elicited from a Cx36ko mouse by green flashes immediately (0 min, red traces) and 15 min after the onset (black traces) of the adapting background light. **(B)** Averaged intensity-response relations at 0, 5, 10, and 15 min after the onset of adapting light. Data were fit with a Naka-Rushton equation, with parameters of K shown in **(C)** and R_max_ shown in **(D)**. Dashed lines denote data from WT mice replotted from Figure 2. **p < 0.01; ***p < 0.001.

As the R_max_ ERG responses to green and UV flashes exhibited similar amplitude enhancements, we used the green flash responses to study the mechanism of this amplitude enhancement during the light-adaption. It has been suggested that light-adaptation mediated alterations in rod-cone photoreceptor coupling contribute to the ERG amplitude enhancement during light adaptation (Heikkinen et al., 2011; Bush et al., 2019). To test this hypothesis, we examined the effects of background light adaptation on photopic ERG responses from connexin36 knock out (Cx36KO) mice which lack gap-junctions between photoreceptors in the retina (Güldenagel et al., 2001; Deans et al., 2002; Asteriti et al., 2017). Figure 3A shows ERG waveforms to green flashes at the onset (0 min) and 15 min after background light adaptation. In contrast to wildtype mice (Figure 1A), 15 min light adaptation in the Cx36KO mouse had minimal effects on the ERG responses elicited by high-intensity light flashes (10, 30, and 100 cd.s/m^2^), while the responses to dim flashes (0.3, 1, and 3 cd.s/m^2^) were reduced indicating desensitization by light adaptation. Averaged intensity-response relations obtained after 0, 5, 10, and 15 min light adaptation are shown in Figure 3B. Continuous curves are shown fitted to the Naka-Rushton equation, with values of K and R_max_ shown in Figures 3C,D, respectively. Values for WT controls are re-plotted as dashed lines from those shown in Figures 2C,D. Compared with wild-type (WT) mice, light adaptation had similar effects in reducing sensitivity (K) of the photopic ERG response in the Cx36KO mouse. There is no statistical difference in K values between WT and Cx36KO mice at any time points during light adaptation (Figure 3C). On the other hand, connexin 36 mutation eliminated the light adaptation-induced enhancement of the maximum amplitude (R_max_; Figure 3D) when compared to the WT mouse.

**Figure 4:**
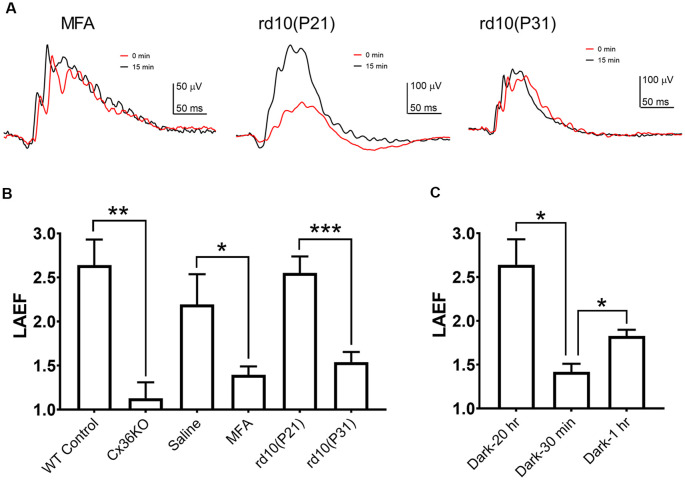
B-wave light adaptation enhancement factor (LAEF). LAEF is defined as the ratio of maximum amplitude elicited after 15 min light adaptation to the amplitude observed at the onset of the adapting light. **(A)** Examples of ERG waveform elicited by 100 cd.s/m^2^ flashes at 0 and 15 min of light adaptation for mice intravitreally injected with meclofenamic acid (MFA), rd10 at P21, and rd10 at P31. **(B)** LAEF obtained under each condition: control, *n* = 5; Cx36KO, *n* = 6; MFA, *n* = 5; P21 rd10, *n* = 8; P31 rd10, *n* = 8. **(C)** Effect of the dark-adaptation duration on LAEF: 20 h (same as control in **A**), *n* = 5; 30 min, *n* = 4; 1 h, *n* = 3. *p < 0.05; **p < 0.01; ***p < 0.001.

To quantify the ERG b-wave amplitude enhancement during light adaptation, we calculated the ratio of the b-wave amplitudes recorded after 15 min light adaptation to those observed at the onset of adapting light, termed the light adaptation enhancement factor LAEF (as previously shown in Figure 2A). For wildtype C57b/6J mice, LAEF averaged 2.64 ± 0.29 (*n* = 5). In Cx36KO mice, light adaptation had little effect on b-wave amplitudes, with an average LAEF of 1.136 ± 0.18 (*n* = 6; Figure 4B). Similarly, intravitreal injection of MFA, a gap junction blocker, also significantly reduced the effects of light adaptation on ERG b-wave amplitudes in WT mice, with LAEF of 1.42 ± 0.28 (*n* = 5) which was significantly different (*p* < 0.05) than the LAEF of saline-injected controls (LAEF = 2.23 + 0.33, *n* = 4; Figure 4B). The ERG waveforms from a mouse intravitreally injected with MFA to 100 cd.s/m^2^ flashes are shown in Figure 4A (left panel) at the onset (0 min) and 15 min after background light adaptation. On the other hand, no statistical difference in the LAEF values was observed between saline-injected and un-injected controls (*p* = 0.56; Figure 4B). Also, the ERG recorded from MFA-injected mice showed significantly larger amplitude (107.9 ± 7.3 μV, *p* < 0.01) than those of saline-injected controls (70.3 ± 7.7 μV).

### Effects of Rod Photoreceptor Degeneration on Mouse Photopic ERG

If rod-cone photoreceptor coupling contributes to the changes observed on the mouse photopic ERG during light adaption, a reduction of gap-junctions such as during rod photoreceptor degeneration should also alter the effects of light adaptation on the photopic ERG responses. We examined the effects of light adaptation on photopic ERG responses of rd10 mice, a commonly used mouse model for retinal degeneration (Chang et al., 2002; Zhao et al., 2015; Li et al., 2018). Example of ERG waveforms from an rd10 mouse at P21 (middle panel) and P31 (right panel) to 100 cd.s/m^2^ flashes at the onset (0 min) and 15 min after background light adaptation are shown in Figure 4A. At P21 in rd10 mice, large portions of rod photoreceptor persist in the retina (Zhao et al., 2015; Li et al., 2018), and the photopic ERG exhibited a similar b-wave enhancement as wildtype control, with LAEF of 2.55 ± 0.19 (*n* = 8, *p* = 0.99; Figure 4B). However, at P31, most of the rod photoreceptors in rd10 mice retina have degenerated (Zhao et al., 2015; Li et al., 2018), and a much reduced b-wave enhancement was observed (LAEF of 1.54 ± 0.12, *p* < 0.001; Figure 4B) suggesting the LAEF is dependent on the number of intact gap junctions between rod and cone photoreceptors in the retina. Consistent with this notion, the b-wave amplitudes of the photopic ERG at the onset of light adaptation were larger for P31 rd10 mice (139.3 ± 19.1 μV) than the responses elicited at P21 (113.3 ± 16.7 μV), although the difference is not statistically significant (*p* = 0.16). After 15 min light adaptation, b-wave amplitudes at P31 were smaller (227.4 ± 24.9 μV, *p* = 0.17) than the responses at P21 (259.5 ± 21.1 μV), most likely due to the degeneration of cone photoreceptors.

### Effects of the Dark-Adaptation Duration on the Mouse Photopic ERG Response

As the modulation of the photoreceptor gap junction has a slow time course, we investigated the effects of different dark-adaptation durations on the LAEF. For the control mice shown in the above section, they were dark-adapted overnight (about 20 h). Light-adaptation enhanced the b-wave amplitudes by 2.64 ± 0.29 fold (*n* = 5). A much smaller LAEF of the B-wave was observed with mice using a shorter dark-adaptation time (Figure 4C). When mice were dark-adapted for only 30 min before ERG recording, the LAEF = 1.55 ± 0.42 (*n* = 4). This reduction is statistically significant (*p* < 0.05). Increasing the dark-adaptation time also increased the light-adaptation enhancement (LAEF). After 1 h dark-adaptation, the LAEF increased to 1.83 ± 0.07 (*n* = 3, *p* < 0.05), but was still much smaller than the value observed after over-night dark-adaptation (Figure 4C). One-way ANOVA analysis of the combined dataset indicates the LAEF is significantly dependent on dark-adaptation time (*p* < 0.05).

### Effects of Gap-Junction Coupling on Flicker ERG

**Figure 5:**
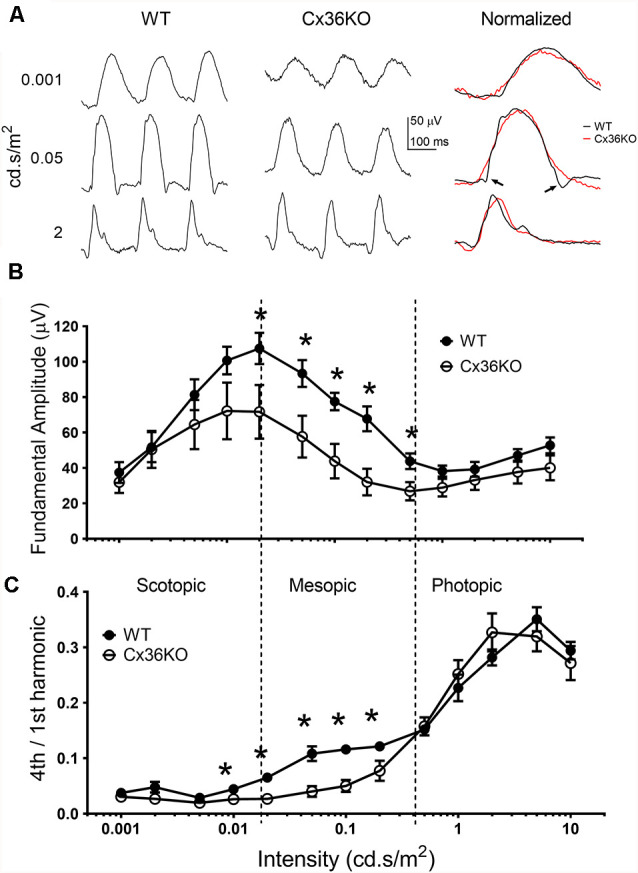
Flicker ERG responses. ERG responses elicited with 6 Hz flash flickering stimulus in the wildtype and Cx36KO mice. **(A)** Examples of the flicker ERG waveform at 3 stimulus intensities for a WT mouse (left panel), and a Cx36KO mouse (middle panel). Amplitude normalized waveforms are shown on the right panel, illustrating the narrower response waveform for WT (pointed by arrows) than those of the Cx36KO elicited by mesopic light stimulus (middle row). **(B)** The amplitude of the fundamental (6 Hz) response. **(C)** The ratio of the 4th harmonic (24 Hz) to fundamental response amplitudes. While both the scotopic and photopic flash intensities showed a similar 4th harmonic component for WT (*n* = 6) and Cx36KO (*n* = 4) mice, there is a much higher 4th harmonic component for WT to mesopic light intensities than in Cx36KO mice. *p < 0.05.

Rod-cone coupling serves as a secondary rod signaling pathway in the retina. We used pulse flicker ERG to investigate how the temporal properties of the visual system were affected by photoreceptor coupling. As described previously (Qian and Shah, 2011), a single pulse flicker stimulus is composed of a series of harmonics, each with equal energy. Therefore, a single pulse flicker stimulus could provide a frequency-response relationship for ERG responses less than 30 Hz. Figure 5A illustrates examples of flicker ERG waveforms to 3 flash intensities for a WT (left panel) and a Cx36KO (middle panel) mouse. To compare the shape of ERG waveform, amplitude normalized flicker ERG waveforms are shown on the right panel of Figure 5A. For responses elicited by either dim (top row) or bright (bottom row) flashes, ERG waveforms from WT and Cx36KO mice were very similar. On the other hand, for the responses elicited with mesopic flashes (middle row), the flicker ERG recorded from WT (pointed by arrows) had much narrower waveform than those recorded from Cx36KO mice.

To better quantitate flicker ERG responses, frequency component analysis of the flicker ERG was performed (Qian and Shah, 2011). Intensity-response relations of the fundamental amplitude elicited by the 6 Hz pulse rate with increasing green LED flash intensities from wildtype and Cx36KO mice are shown in Figure 5B. Similar to their flash ERG differences from WT (Figure 3D), the Cx36KO mice have somewhat smaller flicker ERG response amplitudes at different adapting backgrounds. The intensity-response relationship for both mice exhibited a similar pattern and can be divided into three regions. For flash intensities in the scotopic region (less than 0.02 cd.s/m^2^), response amplitudes increased with the flash intensity and the fundamental amplitudes peaked at 0.02 cd.s/m^2^, indicating activation of the primary rod pathway in the retina. For flash intensities in the mesopic region (0.02–0.5 cd.s/m^2^), response amplitudes decrease with flash intensity, indicating gradual saturation of the primary rod pathway. Here the response of the WT mice was larger than the Cx36KO. For flash intensities in the photopic region (>0.5 cd.s/m^2^), the flicker responses again increase with stimulus intensity, indicating the activation of cone pathways in the retina (Lei, 2012). We used the ratio of the fourth harmonic (24 Hz response) to the fundamental as an index to probe the temporal property of flicker ERG response elicited by various stimulus intensities, and the results are shown in Figure 5C. The ratios were much smaller for the responses elicited with scotopic stimuli (all less than 0.05), whereas those elicited by photopic stimuli were much higher (all larger than 0.2), consistent with cone pathways having much better temporal responses than the primary rod pathway in the retina. In addition, Cx36KO and WT mice had similar harmonic ratio values for light stimuli in the scotopic and photopic regions, indicating similar primary rod and cone pathways are used in these two mice. On the other hand, for light stimuli in the mesopic region, the ratios were consistently higher in the WT mice than those from the Cx36KO mice, indicating the secondary rod signal pathway *via* rod-cone coupling provided enhanced temporal responses for rod signal in the visual system.

### Effects of Background Adaptation on Oscillatory Potentials

**Figure 6:**
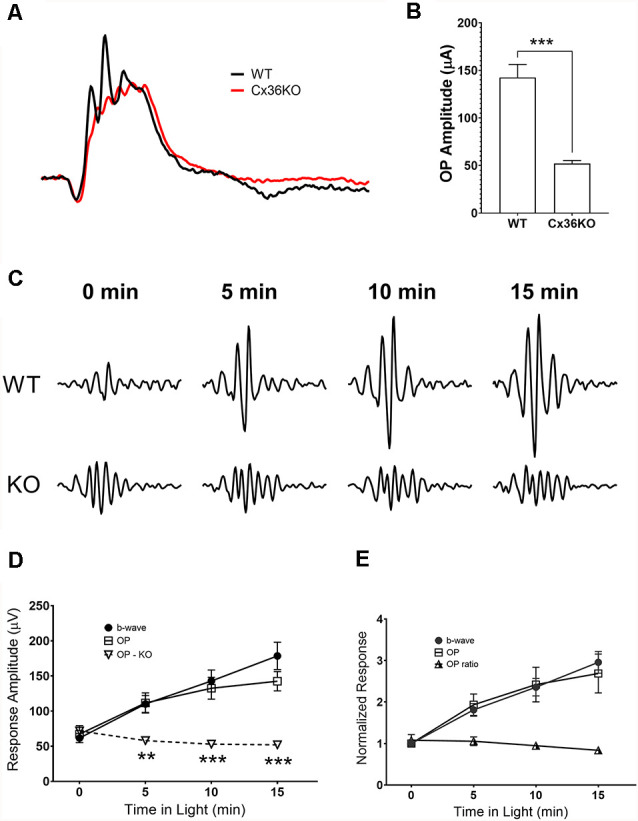
Effects of light adaptation on oscillatory potential (OP) amplitudes. **(A)** Comparison of the ERG waveforms elicited by 100 cd.s/m^2^ flashes and normalized to their respective b-wave amplitude from a WT and a Cx36KO mouse, illustrating the difference in the amplitudes of OPs in responses. **(B)** Averaged OP amplitudes (summed from the first six wavelets) for responses elicited from WT (*n* = 5) and Cx36KO mice (*n* = 6). **(C)** Examples of a WT and a Cx36KO mouse OP waveform digitally isolated from ERG responses at 0, 5, 10, and 15 min after the onset of the adapting light. **(D)** Averaged amplitudes of the ERG b-wave and OPs of WT and the OPs of Cx36KO during light-adaptation (*n* = 5). **p < 0.01; ***p < 0.001 for the difference of OP amplitudes between WT and Cx36KO mice. **(E)** ERG b-wave and OPs of WT mice normalized to value at the time of adapting light onset. Ratios of OP to b-wave amplitudes are also plotted.

Comparing photopic ERG waveforms recorded from wildtype and Cx36KO mice, examples of their normalized responses shown in Figure 6A, one of the main differences is the amplitude of OP wavelets superimposed on their ERG b-wave. A band-pass digitally filtered mouse photopic ERG responses to 100 cd.s/m^2^ green flashes were used to investigate OPs in the ERG waveform. Responses to maximum stimulus intensity were chosen to avoid complications of both sensitivity and amplitude changes during light-adaptation. Averaged OPs summed from 6 wavelets for wildtype and Cx36KO mice after 15 min light adaptation are shown as a bar graph in Figure 6B. ERG responses from wildtype mice had significantly higher amounts of OPs than mice lacking connexin36 (*p* < 0.001). This difference persisted even after normalization to their b-wave amplitudes (0.84 ± 0.07 for WT and 0.32 ± 0.07 for Cx36KO, *p* < 0.0001).

For WT mice, OP amplitudes also increase with light adaptation. Figure 6C illustrates examples of the OP waveform obtained from a WT and a Cx36KO mouse recorded at 0, 5, 10, and 15 min of light adaptation. Summed peak-to-peak amplitudes of the first four OP peaks were plotted in Figure 6D; along with measured b-wave amplitudes for WT. For WT mice, OP responses showed a similar trend of gradual increase in amplitude during light adaptation, whereas OP responses from Cx36KO mice kept at a relatively constant level during light adaptation. Figure 6E plots WT responses normalized to their value at the time of background onset (*t* = 0), again illustrate the similar effects of light adaptation on OP and b-wave amplitudes in WT. Consequently, light adaptation had little impact on the ratios of summed OP amplitudes to the b-wave in WT mice. As OPs of the flash ERG have been shown to arise within the proximal retina due to the participation of inhibitory inner retinal circuity (Wachtmeister and Dowling, 1978; Wachtmeister, 1998, 2001), these results suggest that background adaptation has insignificant additional effects on inner retinal responses as measured by OPs.

## Discussion

In this study, we investigated the effects of rod-saturating background adaptation lights on the mouse photopic ERG. Our results on the ERG b-wave indicate that during the time of light adaptation there is a gradual reduction in its sensitivity (K) and an enhancement of the response amplitude (R_max_; Figures 1, 2). With the exception for a small proportion of genuine S cones (about 5% of total cone population), the majority of mouse cone photoreceptors (M cones) express both UV and green opsins as revealed by immunostaining and electrophysiological recordings (Lyubarsky et al., 1999; Applebury et al., 2000; Haverkamp et al., 2005; Nikonov et al., 2005). Therefore, sensitivity reduction was only seen for the response elicited by green light but not for the response elicited by UV light, indicating there is minimal cross-talk between the green and UV cone opsin-mediated signaling pathways in M cones in the mouse retina. On the other hand, both green and UV ERG responses were similarly enhanced during light adaptation, suggesting a common mechanism for photopic ERG amplitude enhancement may be involved. Also, the OPs of the photopic ERG followed a similar time course of amplitude enhancement with adaptation as the b-wave (Figure 6), suggesting modulation of photopic ERG response by background light-adaptation is dominated by alterations of these responses occurring in the outer retina.

It has been suggested that light-induced alterations in rod-cone coupling in the retina contribute to photopic ERG response changes during light-adaptation (Heikkinen et al., 2011; Bush et al., 2019). However, gap-junctional communications are widely distributed in the mammalian retina (O’Brien and Bloomfield, 2018). In addition to forming gap junctions between photoreceptors, Cx36 is also expressed in AII amacrine cells and mediates inter-AII amacrine cell coupling and coupling between AII cells and ON-bipolar cells. Electrical coupling between AII amacrine cells to cone bipolar cells serve as the primary rod signal pathway (Hartveit and Veruki, 2012). Besides, MFA is also a non-selective gap junction blocker. Although this study did not explicitly provide direct evidence for the role of rod-cone coupling, our results are consistent with the hypothesis that alteration in photoreceptor electrical communication modulates photopic ERG response changes during light-adaptation.

After prolonged dark adaptation, gap-junctions between the rod and cone photoreceptors are open, which provide electrical communications between both types of photoreceptors (Li et al., 2013; O’Brien, 2019). In the dark, photo-responses initiated at cone photoreceptors would shunt off to rods and only a fraction of the signal would be passed onto the inner retina. During light-adaptation, the gap-junctions between rod and cone photoreceptors gradually close, and more cone photoresponse feeds into the inner retinal neurons. In this study, we provide further support for this hypothesis (Figures 3, 4). First, Cx36KO mice, in which gap-junctions among photoreceptors are absent (Güldenagel et al., 2001; Deans et al., 2002; Abd-El-Barr et al., 2009), lack the enhancement of photopic ERG response during light adaptation. Second, enhancement of the photopic ERG responses was significantly reduced by intraocular injection of MFA, a gap junction blocker of rod-cone coupling. Third, the capacity of the light-adaptation induced (LAEF) photopic ERG enhancement is progressively diminished in rd10 mice when there is a loss of a significant portion of the rod photoreceptors. It is interesting to note that although rod photoreceptors have already started to degenerate at P21 in rd10 retina (Zhao et al., 2015; Li et al., 2018), a similar LAEF was observed as WT mice (Figure 4A). It is likely that only the first ring of rod photoreceptors immediately next to cones could act as a sink for cone photo-responses. Therefore, even though a large number of rods degenerated at P21 for rd10 retina, there are still a significant amount of rods immediately next to every cone photoreceptor as in WT mice. At P31, rod photoreceptors become severely degenerated in rd10 mice, and the LAEF is significantly reduced.

Light-induced modulation of photoreceptor coupling is mediated by alteration of the phosphorylation of connexin 36 proteins in these cells (Li et al., 2013; O’Brien, 2019). The time course of such phosphorylation changes is slow. Similarly, we also noticed slow changes of the LAEF during dark adaptation. After 1-h dark-adaptation, LAEF is only about half of that observed after over-night dark-adaption in WT mice. On the other hand, it is possible de-phosphorylation by an as yet unidentified phosphatase could un-couple rod-cone gap junctions with a relatively faster time course (O’Brien, 2019), as significant changes on photopic ERG responses are observed during 15 min of light adaptation (Figures 1, 2).

Rod-cone coupling provides the secondary rod signal pathway in the mammalian retina (Deans et al., 2002; Völgyi et al., 2004; Fain and Sampath, 2018). This secondary rod pathway enables the rod signal to utilize the fast-responding cone circuits in the inner retina (Nelson, 1977). Consequently, the visual system exhibits higher temporal responses under the mesopic condition with this pathway intact than using a simple mixture of rod and cone pathways (Figure 5). The activity of this secondary rod pathway has also been shown on human flicker ERG responses (Bijveld et al., 2011; Park et al., 2015). Also, the secondary rod pathway feeds to a subset of retinal ganglion cells and provides enhanced light sensitivity under mesopic conditions (Völgyi et al., 2004).

One of the prominent differences in ERG waveform elicited from WT and Cx36KO is a significant reduction of OPs in KO mice (Figures 6A,B). OPs are high-frequency oscillation riding on ERG b-wave that is mediated by inhibitory circuitries in the inner retina (Wachtmeister and Dowling, 1978; Wachtmeister, 1998, 2001). It is interesting to note that Cx36KO mice also lack high-frequency oscillation (gamma wave) in the brain (Hormuzdi et al., 2001). On the other hand, for ERG responses elicited from WT mice, the fraction of OPs in the waveform does not change significantly during light adaptation (Figure 6). There are several possibilities to account for these observed results. The light adaptation-induced reduction of gap junctional communication may not be the same as complete removal of electrical coupling among neurons as in KO mice. Also, KO mice congenitally lack gap junctional communication, which might alter the development of neuronal circuity and modify the pathway that generates OPs for flash ERG.

In this study, we have provided a novel approach to noninvasively measure the dynamics of rod-cone gap junctional coupling in intact eyes. Our study provides additional evidence to support that changes of the photopic ERG observed during light-adaption are mediated largely by the modulation of rod-cone coupling. The amount of photopic ERG amplitude enhancement during light-adaptation could be used as an clinical index to noninvasively study the dynamics of rod-cone gap junction coupling in intact eyes.

## Data Availability Statement

The raw data supporting the conclusions of this article will be made available by the authors, without undue reservation.

## Ethics Statement

The animal study was reviewed and approved by National Eye Institute (NEI) Animal Care and Use Committee.

## Author Contributions

YL conceived the study, performed experiments, analyzed the data, and drafted the manuscript for intellectual content. EC assisted with experimental design and completion, analyzed the data, and drafted the manuscript for intellectual content. HQ conceived the study, assisted with experimental design and completion, analyzed the data, and drafted the manuscript for intellectual content.

## Disclaimer

The mention of commercial products, their sources, or their use in connection with material reported herein is not to be construed as either an actual or implied endorsement of such products by the Department of Health and Human Services.

## Conflict of Interest

The authors declare that the research was conducted in the absence of any commercial or financial relationships that could be construed as a potential conflict of interest.
